# Exploring postoperative atrial fibrillation after non-cardiac surgery: mechanisms, risk factors, and prevention strategies

**DOI:** 10.3389/fcvm.2023.1273547

**Published:** 2023-12-07

**Authors:** Shengjie Jiang, Xiaozu Liao, Yong Chen, Binfei Li

**Affiliations:** Department of Anesthesiology, Zhongshan City People’s Hospital, Zhongshan, Guangdong, China

**Keywords:** non-cardiac surgery, postoperative atrial fibrillation, mechanisms, beta-blockers, NT-ProBNP

## Abstract

Atrial fibrillation (AF) stands as the most prevalent persistent arrhythmia and a common complication after surgical procedures. Although the majority of non-cardiac surgery patients experience postoperative AF (POAF) and the condition is typically self-limited and asymptomatic, its detrimental impact on patient outcomes, prolonged hospitalization, and heightened risk of stroke and overall mortality has become increasingly evident. Of significant concern, POAF emerges as a noteworthy risk factor for stroke, myocardial infarction, and mortality in comparison to patients with non-surgical atrial fibrillation. Multiple studies have corroborated the association between POAF and an elevated risk of stroke and mortality. The development of postoperative atrial fibrillation is multifactorial, with the inflammatory response being a primary contributor; additionally, factors such as hypovolemia, intraoperative hypotension, anemia, trauma, and pain can trigger POAF. Risk factors for POAF in non-cardiac surgery primarily relate to age, hypertension, obesity, prior cardiac disease, obstructive sleep apnea, and male sex. Prophylactic treatment with β-blockers, amiodarone, or magnesium has demonstrated efficacy, but further trials are warranted, especially in high-risk populations. This review provides an account of the incidence rate, pathophysiology, and prognosis of atrial fibrillation after non-cardiac surgery, elucidates the underlying mechanisms of its occurrence, and explores various preventive strategies investigated in this domain.

## Introduction

Atrial fibrillation (AF) stands as the most prevalent persistent arrhythmia encountered in both clinical practice and as a common complication following surgical interventions. AF assumes the highest frequency among all rhythm disturbances observed in clinical settings (1). Postoperative atrial fibrillation (POAF), delineated as the emergence of new-onset AF within a 30-day timeframe after surgery, is typically identified through the application of electrocardiography (ECG) techniques, encompassing either a single recording or a comprehensive 24-h monitoring session (2). Estimations suggest that the collective magnitude of major surgical procedures performed on a global scale annually exceeds 300 million cases, constituting approximately 5% of the global population. Notably, the substantial majority, around 85%, pertain to non-cardiac surgery (NCS) (3, 4). Moreover, it is worth highlighting that adults aged 65 years or older account for a notable 37% of all inpatient procedures in the United States, and with advancing age, the cardiovascular risk profile elevates accordingly. Furthermore, the data also imply a correlation between increasing age and augmented susceptibility to postoperative complications (5).

Historically, the occurrence of POAF in NCS patients was mistakenly perceived as a relatively benign complication, primarily due to its self-limiting nature and asymptomatic presentation. However, recent investigations have illuminated the detrimental impact of POAF, which encompasses hemodynamic instability, prolonged hospitalization, infectious complications, pulmonary complications, heightened risk of stroke, elevated in-hospital mortality rates, and escalated healthcare expenditures (2, 6–8).

We conducted a comprehensive search in the Medline and PubMed databases, employing a combination of search terms including “postoperative atrial fibrillation,” “atrial fibrillation,” “mechanism,” “pathophysiology,” “incidence,” “risk factors,” “predictors,” “prognosis,” “prevention,” and “non-cardiac surgery.” This search encompassed relevant articles published between 1995 and 2023. Furthermore, we meticulously examined the reference lists of each article to identify additional relevant publications. In the context of this review, our objective was to provide insights into the fundamental pathophysiology, predictive determinants, prognostic implications, and strategies for preventing POAF following non-cardiac surgical procedures.

### Incidence

The occurrence of POAF following NCS demonstrates a wide-ranging incidence rate, spanning from 2% to 30%, with a peak manifestation typically observed within 2–4 days postoperatively (9, 10). In a study conducted by Roselli et al., including 608 patients diagnosed with lung cancer, a noteworthy 19% incidence rate of postoperative atrial fibrillation was observed after thoracic surgery (11). In a prospective study including 799 patients afflicted with malignant neoplasms and undergoing NCS, POAF was observed in approximately 10% of the cases (12). Likewise, a retrospective investigation comprising 461 individuals undergoing liver transplantation documented a prevalence rate of POAF at 10.2%. The median onset of POAF occurred approximately 3 days following the transplantation procedure. Notably, among them, patients with POAF exhibited a significantly higher prevalence rate of thromboembolic events than those without POAF (17.0% vs. 3.1%; *P* < 0.001) (13).

A cohort study comprising 1,210 patients undergoing upper gastrointestinal surgery documented the aggregate prevalence rate of POAF at 8.3%. Furthermore, the incidence of POAF exhibited variability contingent upon the extent of the procedure and the specific site of resection. Notably, the highest incidence rate was displayed by complex esophagectomy at 45.5%, followed by elective thoracoabdominal esophagectomy at 17.1% and total pancreatectomy at 16.7%. In the context of multiple organ resection surgeries, the incidence rate of atrial fibrillation was documented at 12.7%, while extended gastrectomy, gastrectomy, pancreatic head resection, and gastric sleeve or gastric bypass resection yielded incidence rates of 7.6%, 5.2%, 5.0%, and 1.9%, respectively (14).

In a retrospective analysis comprising 1,171 patients undergoing surgical procedures related to the lower gastrointestinal tract, a prevalence rate of 4.8% was observed in the development of POAF. Specifically, patients undergoing bowel resection for mesenteric ischemia exhibited the highest incidence rate at 26.92%, followed by cytoreductive surgery (CRS) in combination with abdominal thermochemotherapy at 16.67%. Conversely, abdominal perineal proctocolectomy/resection showed an incidence rate of 4.4%, while left hemicolectomy/sigmoid colectomy/anterior rectal resection and Hartmann surgery demonstrated a notably lower incidence rate of POAF at 3.83% (15). It is worth noting that the true incidence rate of POAF in patients may be underestimated since the majority of included studies relied on POAF reports from databases rather than utilizing continuous ECG monitoring to capture arrhythmias. The incidence of POAF could exhibit variability contingent upon the methodology or frequency of ECG surveillance. Notably, Higuchi et al. reported that 83% of patients with postoperative AF were asymptomatic (12), further suggesting that a significant proportion of POAF episodes may go unnoticed.

### Pathophysiology

The pathogenesis of POAF is intricate and involves a confluence of factors that render the atria susceptible to the initiation and perpetuation of POAF. In the context of cardiac surgery, cardiomyocyte inflammatory signaling plays a pivotal role in the development of POAF, wherein elevated levels of inflammatory markers, including C-reactive protein (CRP), white blood cells (WBCs), and interleukins, are associated with POAF (16, 17). A comparable underlying process may transpire in the setting of non-cardiac surgery. Notably, aside from systemic inflammation, POAF can be incited by local inflammation of structures adjacent to the resection site or even inflammation within the resected pulmonary veins (18). Mechanistically, surgical stress triggers activation of the sympathetic nervous system, thereby augmenting heart rate and catecholamine release, both of which can contribute to the onset of atrial fibrillation. Moreover, various clinical conditions such as hypovolemia, intraoperative hypotension, anemia, trauma, and pain can also influence sympathetic activity and serve as potential triggers for POAF (19).

### Risk factors and predictors

Numerous studies have endeavored to identify potential risk factors and predictors associated with POAF following NCS (Table 1).

**Table 1:** Risk factors and predictors associated with POAF following NCS.

Categories	Factors	Studies
Patient-related	Advanced age	(2, 17, 20–23)
	Heart failure	(2)
	Hypertension	(2, 24, 25)
	COPD	(24)
	Myocardial infarction	(24)
	Left atrial enlargement	(25)
	Male gender	(26, 27)
	Obesity	(28)
	Previous AF episodes	(26)
	Poor physical status	(26)
	OSAS	(29)
Blood volume	Hypovolemia	(30, 31)
	Excessive blood volume	(19)
Surgery related	Surgical approach	(2)
	Glycemic fluctuations	(32)
	Red blood cell transfusion	(33)
	Mechanical ventilation	(34, 35)
Anesthesia management		(36, 37)
Postoperative complications	Sepsis	(2, 13)
	Pneumonia	(2)
	Pleural effusion	(2)
Drug related	Vasopressors	(38)
Natriuretic peptides		(39–44)
Echocardiography		(45–49)

### Patient-related factors

Advanced age has consistently emerged as a significant contributor to the development of POAF (20–23). Amar et al. conducted a study revealing a 1.8-fold increased risk of POAF for every 10-year increment in age (17). Similarly, a meta-analysis by Chebbout et al. involving a large cohort of 52,959 patients demonstrated a positive correlation between age and POAF risk in NCS. Notably, a history of cardiac conditions, particularly congestive heart failure and hypertension, exhibited a significant association with POAF in the aforementioned study (2). Yamashita et al., in another meta-analysis, found that among patients who developed POAF, comorbidities such as heart failure, arterial hypertension, chronic obstructive pulmonary disease (COPD), and myocardial infarction substantially elevated the risk compared to unaffected individuals (24). Furthermore, independent predictors of AF recurrence post-discharge included moderate to severe left atrial enlargement and hypertension, as identified through multivariate analysis (25). Additional factors such as male gender, obesity, previous AF episodes, and poor physical status have been consistently linked to an increased risk of POAF (26–28). Moreover, a multicenter prospective cohort study revealed that unrecognized severe obstructive sleep apnea syndrome (OSAS) significantly heightened the risk of postoperative cardiovascular complications, including AF. Conversely, the type of anesthesia and the use of postoperative opioids did not exert a significant influence on postoperative cardiovascular outcomes, as supported by the study findings (29).

### Blood volume

Hypovolemia, arising from factors such as fluid loss, anesthesia-induced cardiovascular depression, or sepsis, can result in reduced venous return to the right atrium, leading to diminished cardiac output. This hemodynamic alteration triggers the release of catecholamines and can ultimately provoke the onset of atrial fibrillation. Intraoperative mean arterial pressure below 60–70 mmHg is associated with myocardial injury and mortality. The severity and duration of hypotension are determinants of the aforementioned complications (30, 31). It is worth considering that myocardial ischemia, potentially attributable to blood loss or anemia, can also precipitate arrhythmias. Excessive blood volume results in increased intravascular volume, leading to stretching of the right atrium and subsequently triggering atrial fibrillation (19). In the context of pulmonary surgery, vasoconstriction of the pulmonary veins can induce elevated right ventricular pressure, further contributing to atrial stretching (50). Furthermore, in patients with chronic AF, the pulmonary veins have been identified as a significant source of ectopic beats, which can serve as triggers for recurrent AF episodes (51).

### Surgery related factors

POAF is influenced by the surgical approach, with open surgery exhibiting a higher incidence rate than laparoscopic surgery and procedures involving the thoracic region showing a greater propensity for POAF compared to other interventions (2). Perioperative glycemic fluctuations play an independent role as a risk factor for POAF, with pronounced fluctuations being associated with mitochondrial superoxide production, release of inflammatory cytokines, and oxidative damage (32). Red blood cell transfusion has been shown to modulate inflammation by elevating plasma levels of inflammatory markers, thereby increasing the occurrence of postoperative AF (33). A retrospective study identified prolonged mechanical ventilation as an independent risk factor for postoperative arrhythmias, including AF (34). Mechanical ventilation induces changes in intrathoracic pressure, and both intermittent positive-pressure ventilation and positive end-expiratory pressure contribute to elevated right atrial pressure and heightened sympathetic activity, predisposing individuals to POAF (35). The duration of surgery is positively correlated with the incidence of POAF, with longer surgical procedures associated with a higher risk (52).

### Anesthesia management

Most investigations on intraoperative risk factors have focused on surgical aspects, with limited studies exploring the influence of intraoperative anesthesia management. General anesthesia has been associated with a higher incidence rate of AF at 1 year postoperatively than conscious sedation (36). In thoracoscopic lung cancer surgery, combined paravertebral nerve block has shown a reduced incidence rate of POAF when compared to general anesthesia (37). Regarding NCS, there is no significant difference in the incidence rates of POAF between volatile anesthetics and general intravenous anesthesia (53). In a multicenter randomized controlled study, volatile anesthetics demonstrated superior myocardial protection in patients undergoing coronary surgery compared to general intravenous anesthesia (54). However, another study focusing on non-cardiac surgery found no significant myocardial protection provided by volatile anesthetics compared to general intravenous anesthesia (55). Additional research is needed to ascertain whether volatile anesthetics offer cardioprotection in non-cardiac surgical settings.

### Postoperative complications

Postoperative complications, including sepsis, pneumonia, and pleural effusion, demonstrated a significant association with the occurrence of POAF, while sepsis exhibited a notable correlation with an increased incidence of POAF (2). Notably, postoperative sepsis emerged as an independent predictor of POAF, as demonstrated in the study conducted by Koshy et al. (13). Furthermore, a study revealed that a twofold rise in the postoperative leukocyte count compared to the preoperative count was associated with a 3.3-fold increase in the odds of developing POAF (17).

### Drug related factors

Indeed, research has demonstrated an association between elevated postoperative norepinephrine levels, the utilization of vasopressors or positive inotropic agents, and an increased risk of developing POAF. Conversely, the administration of β-blockers for prophylaxis has been shown to reduce the incidence of POAF (38). A retrospective study revealed that isoproterenol anesthesia was associated with a decreased occurrence of new-onset POAF compared to desflurane (56). On the other hand, in a multicenter randomized controlled trial, dexmedetomidine did not significantly impact the incidence of POAF (57). However, another study demonstrated that the intraoperative use of dexmedetomidine led to a reduction in the occurrence of POAF (58), which may be attributed to its antiarrhythmic effects mediated through its sympathetic properties and activation of the vagus nerve.

### Natriuretic peptides

Patients with elevated preoperative B-type natriuretic peptide (BNP) measurements are at an increased risk of developing POAF (39). In a prospective study conducted by Cardinale et al. in 2007, the ability of natriuretic peptide to predict POAF was investigated. NT-proBNP levels were measured in patients 24 h before thoracic surgery and 1 h after surgery. It was observed that patients with an increase in this marker had a significantly higher incidence of POAF than those with normal levels. Both preoperative and postoperative NT-proBNP values demonstrated the ability to predict the occurrence of POAF (40). Another study by Matsuura et al. demonstrated that high preoperative NT-proBNP levels predicted the incidence of POAF in patients undergoing non-extracorporeal coronary artery bypass grafting (OPCAB) (41). A meta-analysis conducted by Cai et al. revealed that natriuretic peptides exhibited a sensitivity of 75% and specificity of 80% for predicting POAF, with a positive likelihood ratio of 3.28. Furthermore, NT-proBNP demonstrated superior predictive power compared to BNP, and postoperative assessment showed greater efficacy than preoperative assessment (42). In a prospective study by Szczeklik et al., higher NT-proBNP values were associated with a greater predictive ability for POAF, and preoperative NT-proBNP levels were independently associated with the development of POAF (43). Gurgo et al. demonstrated that patients who developed POAF had more than two times higher preoperative NT-proBNP levels, and elevated preoperative plasma NT-proBNP levels were linked to the development of POAF in patients undergoing major thoracic surgery (44). Overall, NT-proBNP levels may serve as a useful predictor for identifying patients at higher risk of POAF, enabling proactive administration of prophylactic medication.

### Echocardiography

Left ventricular mitral early transmitral velocity/mitral annular early diastolic velocity (E/e’) calculated by echocardiography has been proposed as an independent predictor of POAF, as demonstrated in a study by Iwata et al. (45). In addition, a study by Brecher et al. revealed that in non-cardiac surgery (NCS), both echocardiographic indices of diastolic dysfunction and an increase in preoperative BNP were predictive of POAF. However, when assessing these factors together, the preoperative left atrial diastolic volume index and the deceleration time of transmitral flow emerged as independent predictors of POAF, while BNP did not retain its independent predictive value (46). Furthermore, a study by Mita et al. indicated that preoperative biventricular diastolic dysfunction in patients undergoing lung surgery was associated with the development of POAF (47). Furthermore, Oh et al. demonstrated that an elevated preoperative left atrial volume index (LAVI) assessed by echocardiography was significantly associated with the occurrence of POAF in NCS (48). Nevertheless, in the investigation conducted by Ai et al., preoperative echocardiographic variables were found to lack predictive value for the occurrence of POAF in non-cardiac thoracic surgery (49). The ability of echocardiography to predict the incidence of POAF in patients undergoing NCS continues to be a subject of controversy.

### Prognosis

Previously, POAF was regarded as a relatively benign condition primarily confined to the postoperative period (59). Consequently, limited attention has been devoted to the prevention and management of POAF by many investigators. However, an expanding body of evidence has underscored the notable recurrence rate and associated complications of POAF. Of particular significance, patients with POAF face an increased risk of stroke, myocardial infarction, and mortality compared to their counterparts without POAF. These findings have prompted a reevaluation of the significance of POAF and have emphasized the importance of proactive measures targeting its prevention and treatment (10, 60).

Recent meta-analyses have also yielded consistent findings: in a comprehensive meta-analysis, patients with POAF exhibited a 62% higher risk of early stroke, a 37% higher risk of long-term stroke (with a long-term stroke rate of 2.4% in POAF patients compared to 0.4% in patients without POAF), and a 44% and 37% higher risk of early and long-term mortality, respectively, when compared with patients without POAF (61). Notably, the risk of stroke or transient ischemic attack (TIA) in patients with POAF was found to be similar to that in patients without surgical atrial fibrillation [with an absolute risk difference (ARD) of 0.1% (95% CI, −2.9% to 3.1%) at 5 years and a hazard ratio (HR) of 1.01 (CI, 0.77–1.32)]. There were no significant differences observed in cardiovascular or all-cause mortality (62). In a study by Koshy et al., patients undergoing non-cardiac surgery were followed for an average of 1.4 years, and it was determined that the risk of stroke associated with POAF was 2.5 times higher than that in patients without POAF (63). Similarly, in the study conducted by Lin et al., POAF was linked to an increased risk of stroke and death, both in the short term and in the long term, in patients undergoing non-cardiac surgery (61). Furthermore, the study by AlTurki et al. revealed a threefold increase in the short-term risk of stroke and a fourfold increase in the long-term risk of stroke and cardiac machine infarction associated with POAF. In addition, POAF was associated with a threefold increase in 30-day all-cause mortality (10). Finally, in a meta-analysis performed by Albini et al., it was demonstrated that POAF was associated with a fourfold increase in the risk of long-term stroke and an approximately 3.6-fold increase in long-term mortality when compared to patients without atrial fibrillation (60).

Lowres et al. performed a comprehensive analysis incorporating systematic review and meta-analysis to explore the frequency of AF recurrence in patients discharged with restored sinus rhythm following their initial episode of POAF. The study revealed a recurrence rate of 28.3% among the patients. Notably, a significant proportion, ranging from 61% to 100%, experienced recurrent AF within 2 years (64). These findings underscore the importance of considering POAF as more than a transient complication following surgery. It highlights the necessity for enhanced and meticulous monitoring of patients to enable early identification of recurrent POAF. Prompt intervention should be initiated to mitigate the risks of stroke and myocardial infarction, ultimately ensuring that a greater number of patients can derive substantial benefits from such proactive management strategies.

### Prevention

Considering the implications of POAF in relation to morbidity, mortality, and financial burdens, various preventive strategies designed to diminish its occurrence have been scrutinized. Various pharmaceutical agents have been employed to prevent POAF; nevertheless, the role of pharmacologic prevention remains a subject of controversy (Table 2). The overarching conclusion is that the implementation of preventive measures appears to yield beneficial outcomes. It is crucial to acknowledge that the efficacy of the various studied preventive approaches is not uniform.

**Table 2:** Common pharmaceuticals to prevent POAF.

Pharmaceuticals	Helpful	Studies
Beta-blockers	Yes	(65–72)
Amiodarone	Yes	(73–81)
Magnesium	Yes	(74, 82–87)
Calcium channel blockers	Yes	(72, 74, 88)
	No	(89–91)
Statins	Yes	(72, 92–94)

### Beta-blockers

Among these agents, metoprolol, a β-blocker, has been extensively studied and is considered one of the primary interventions for preventing POAF. Preoperative administration of β-blockers has demonstrated efficacy in reducing the incidence of POAF (65–68). This was supported by a meta-analysis conducted by Zhao et al., focusing on patients undergoing general thoracic surgery, which confirmed the superior preventive benefits of β-blockers in this setting (69). The findings were also corroborated by another meta-analysis by Wang et al. (70). In specific high-risk patients undergoing NCS, the use of perioperative low-dose bisoprolol patches proved effective in preventing POAF (71). Although β-blockers showed no significant impact on respiratory complications or hypotension, it is important to note that they may potentially increase the risk of bradycardia (72). While this observation implies that preoperative metoprolol administration may deter the onset of POAF, it is crucial to avoid isolating these findings. It is essential to recognize that this intervention is associated with an elevated risk of mortality and stroke.

### Amiodarone

In a systematic review and meta-analysis, amiodarone emerged as the most efficacious agent for preventing POAF in lung surgery, demonstrating a remarkable reduction in AF incidence rate from 39.2% to 8.3% without significant adverse effects (73). The effectiveness and safety of amiodarone in preventing POAF during lung surgery have been extensively documented in a study by Riber et al. (74). Riber et al. conducted two separate investigations involving lung cancer patients undergoing thoracic surgery, both revealing significant reductions in POAF incidence with amiodarone usage, although hospitalization time and costs were not significantly affected (75, 76). Another meta-analysis indicated that amiodarone (administered orally or intravenously) was as effective as β-blockers in reducing POAF (77), while the combination of both agents yielded even more favorable outcomes than β-blockers alone (78). However, the safety of prophylactic amiodarone remains a matter of controversy. Lower cumulative doses of amiodarone (<3,000 mg) demonstrated efficacy with fewer adverse events (75, 79, 80). A retrospective study by Tisdale et al. found that although amiodarone significantly reduced the incidence of POAF, it did not impact the length of hospitalization while potentially leading to hypotension, bradycardia, and prolongation of the corrected QT interval (81). This highlights potential side effects associated with amiodarone usage for POAF prevention, prompting cautious consideration, and it is therefore recommended primarily for high-risk patient populations.

### Magnesium

Plasma electrolyte calibration represents a promising preventive approach for POAF, with particular attention given to magnesium as a subject of investigation. Preoperative prophylaxis using magnesium sulfate has been shown to effectively reduce the risk of POAF following pneumonectomy (74). In a comparative trial between amiodarone, control, and magnesium groups, magnesium was found to be less effective than amiodarone but exhibited better tolerability (POAF incidence rate of 20.5% in the control group, 10% in the amiodarone group, and 12.5% in the magnesium group) (82). Two separate meta-analyses further confirmed magnesium's efficacy in reducing POAF incidence after cardiac surgery (83, 84). As for its potential to prevent POAF after non-cardiothoracic surgery, we refer to the study conducted by Terzi et al. in 1996, which demonstrated that magnesium infusion successfully decreased the incidence of POAF in such cases (85). Subsequent studies have similarly supported these findings (86, 87).

### Calcium channel blockers

Preoperative administration of calcium channel blockers has been identified as an effective prophylactic strategy in reducing the risk of POAF following pulmonary resection (74) without concomitantly increasing the likelihood of respiratory complications, hypotension, or bradycardia (72). In a study conducted by Van Mieghem et al., verapamil demonstrated superiority over placebo in decreasing the incidence of POAF after pulmonary surgery; however, it also induced bradycardia and hypotension in a significant number of patients (88). Nevertheless, there exist several studies with conflicting outcomes; for instance, diltiazem prophylaxis did not exhibit efficacy in reducing the occurrence of POAF after thoracoscopic lobectomy (89, 90). Furthermore, a recent investigation by Hochreiter et al. revealed that diltiazem prophylaxis failed to reduce the incidence of POAF after thoracoabdominal esophagectomy and instead led to increased occurrences of bradycardia and norepinephrine requirements (91). Moreover, calcium channel blockers were found to elevate the risk of bradycardia and hypotension, as documented in a study by Riber et al. (74).

### Statins

Statins possess anti-inflammatory and antioxidant properties, which make them potential agents for reducing the incidence of POAF (72). A meta-analysis conducted by Oesterle et al. provided evidence that statin prophylaxis effectively reduces the occurrence of POAF in non-cardiac surgeries, with a relatively low overall risk of short-term adverse events (72). Similarly, in a retrospective study involving 370,447 patients who underwent non-cardiac surgeries, Bhave et al. found that statin prophylaxis was associated with a decreased incidence of POAF (92). These findings were further supported by a meta-analysis carried out by Ma et al. (93). In a study involving patients undergoing pneumonectomy conducted by Amar et al., atorvastatin-treated patients demonstrated a trend toward reduced POAF and other postoperative complications, although the results did not reach statistical significance (94).

### Anesthesia management

In a study conducted by Komatsu et al., it was observed that the use of epidural analgesia did not yield a statistically significant reduction in the incidence of POAF compared to general anesthesia during pulmonary resection. However, patients receiving epidural analgesia tended to experience fewer postoperative cardiovascular complications (95). Similarly, in patients undergoing major abdominal surgery with a history of myocardial injury, thoracic segment epidural analgesia was found to reduce the occurrence of arrhythmias, including AF (96). Intraoperative measures aimed at avoiding electrolyte imbalances, hypovolemia, hypotension, anemia, and pain may contribute to the prevention of POAF.

Additional investigations are warranted to ascertain the optimal and efficacious strategies for preventing AF following NCS. Furthermore, there is a need to identify the patient subgroups most likely to derive substantial benefits from AF preventive measures.

## Conclusions

POAF represents a prevalent complication in non-cardiac surgeries, and this article provides a concise analysis of its incidence, pathophysiology, mechanisms, prognosis, prediction, and prevention. For patients at elevated risk of POAF, meticulous electrocardiogram monitoring is imperative for early detection. Anesthesiologists should exercise particular vigilance when dealing with the elderly, obese, or those with preoperative comorbidities like cardiovascular disease, COPD, and OSAS, especially male patients. Cautionary attention is warranted for patients with high intraoperative blood transfusions and significant blood glucose fluctuations, necessitating vigilant anesthesia management. Perioperative risk factors, including serum potassium, serum magnesium, cardiac ultrasound, and inflammatory factors, should be closely monitored, and proactive measures should be taken to prevent POAF in patients with critical values.

The delineation of POAF risk factors is becoming clearer, and prophylactic treatments show promise in reducing its incidence. Future investigations should evaluate comprehensive treatment approaches for POAF in high-risk patients, tailoring interventions to individual patients, and reducing the overall occurrence of POAF to mitigate long-term complications. Moreover, the effects of certain medications on POAF remain uncertain, necessitating further exploration into their impact, optimal dosages, and administration methods.

### Limitations

It is essential to acknowledge the limitations of this study. The presence of publication bias might have impacted the inclinations of the conclusions presented in this paper. In addition, an in-depth exploration of individual patient attributes, encompassing elements like genetics and comorbidities, which contribute to the predisposition to POAF, was not sufficiently detailed and should be subject to more comprehensive examination in future research endeavors.
